# The patterns, trends and major risk factors of suicide among Indian adolescents – a scoping review

**DOI:** 10.1186/s12888-023-05447-8

**Published:** 2024-01-09

**Authors:** Rachel Elizabeth Senapati, Susangita Jena, Jayashree Parida, Arpita Panda, Prasanna Kumar Patra, Sanghamitra Pati, Harpreet Kaur, Subhendu Kumar Acharya

**Affiliations:** 1 ICMR- Regional Medical Research Centre, NALCO Nagar, Chandrasekharpur, Bhubaneswar, Odisha 751023 India; 2 Department of Anthropology, Utkal University, Bhubaneswar, Odisha India; 3 Division of Epidemiology and Communicable Diseases (ECD-Tribal Health), Indian Council of Medical Research (ICMR), New Delhi, India

**Keywords:** Mental health, Self-harm, Suicidal ideation, Depression, Indian adolescents, Review-of-literature

## Abstract

**Background:**

Adolescence is an essential stage for the development of mental health, and suicide is among the leading cause of mortality for adolescents around the world. In India, the suicide rate among adolescents has been increasing in recent years. The scoping review was conducted to map the evidence and address gaps by examining the existing pattern, and trends, and identify the major risk factors of suicide among Indian adolescents.

**Methods:**

The study was conducted as per the Arksey and O’Malley scoping review framework and the Joanna Briggs Institute Reviewers’ manual. The systematic search was performed using electronic databases such as PubMed, Google Scholar, EMBASE, and PsycINFO, by using specific keywords. After the screening, 35 articles were identified according to the inclusion criteria.

**Results:**

The evidence on the trends of suicide among adolescents showed that the suicide rate has shown an alarming increase in recent years. The evidence pattern showed that hanging and poisoning were the commonly selected methods used by adolescents. The most commonly reported risk factors were mental health problems (54.28%), negative or traumatic familiar issues (34.28%), academic stress (22.85%), social/lifestyle factors (20%), violence (22.85%), economic distresses (8.75%), relationship factor (8.75%).

**Conclusion:**

By synthesizing and summarising the patterns, trends, and key risk factors of suicide among Indian adolescents, this scoping review provides a broad understanding of the literature already in existence. In order to effectively tackle these issues, the finding highlights the urgent need for extensive and targeted suicide prevention measures.

**Supplementary Information:**

The online version contains supplementary material available at 10.1186/s12888-023-05447-8.

## Background

Suicide is the deliberate act of killing own self [1]. Suicidal behavior comprises suicidal ideation, threats or plans of suicide, suicidal attempts, and completed suicide [2]. Studies show that more than 20 suicidal attempts are made within every 40 s out of which at least one person dies [3]. Out of the total number of suicides happening across the world, most of the suicides occur in low -and -middle-income countries [4]. India reported the highest suicide rate in 2021 with 12 suicides for every 100,000 population [5]. Furthermore, this trend, according to Indian National Crime Records Bureau (NCRB) has risen at the rate of 6.2% compared to the year 2020 [5]. Over the past few decades, the suicide rate in India has been increasing rapidly [6]. Adolescent suicides constitute a major proportion of the total number of suicides happening in India every year. Here it is important to mention that the adolescent population in India constitutes one-fifth of its total population and is the largest for any country in the world [7].

Adolescence is defined as the age group ranging between 10–19 years [8]. The period of adolescence comprises a lot of rapidly occurring changes in the physical, social, emotional, cognitive, and intellectual domains of an individual; one of the major outcomes of this rapid development among adolescents is risk-taking behaviors [9]. Also, the phase of adolescence marks the remarkable transition from childhood to adulthood and is characterized by increasing responsibilities, expectations, and exploration of self and identity. The adolescents try to figure out their place and role in society and among their peers in terms of their work, education, family, etc., generating a lot of frequent changes and instability in their lives [10]. Hence, lack of experience and inability to handle all these changes together brings several mental health issues that increasingly affect young people [1, 2]. The 2019 worldwide statistics show that an estimated 166 million adolescents (89 million boys and 77 million girls) had mental health conditions, which means one in seven adolescents experience mental health issues [11]. Studies show that adolescents in India have shown high vulnerability to mental health issues and have consistently been highly affected by suicidal tendencies [12–14]. It has been observed that suicide is the 4th leading cause of death among the late adolescent age groups (15–19 years) in India [4]. According to the NCRB, 2021 report, a total number of 10,730 adolescents (below 18 years of age) died due to suicide [5]. More broadly, mental health conditions are a major risk factor in the adolescent age group and suicide has been at the forefront among all [15].

There have been several studies in different parts of the country on various aspects of suicide in India. Such studies have reported methods, means, reasons, risk factors, and other aspects of suicide [16–18]. So, detailed knowledge about these patterns is important for correct prevention strategies [3]. The risk factors of suicide increase the potential for a person’s suicide or suicidal behavior. Studies show that the interaction between multiple factors like biological, psychological, sociocultural, and family-like factors can cause major risks/reasons for adolescent suicide [19, 20]. The underlying factors like being an adolescent, gender, or ethnicity have been reported as potential to increase the impact of certain risk factors [21]. Also, the year-wise statistics of suicide, particularly among adolescents and youth indicate a crisis in public health that needs to be taken up on an urgent basis with preventive strategies [22].

The present review examined studies published in the past decades and used a systematic approach to identify key themes and trends. Also, the review identified various risk factors connected to suicidal behavior, thoughts, and attempts among Indian adolescents as well as studies that specifically address these issues. This scoping study is crucial because it comprehensively explores the existing literature, mapping of the evidence, analysis of the available information, and identifies research gaps on adolescent suicidal behaviors in India. The results of this study can help to guide interventions and policies targeted at lowering the suicide rate in vulnerable groups.

### The objectives

To map the evidence regarding the patterns and trends of suicide among Indian adolescents.

To evaluate the evidence on the major risk factors influencing the suicidal behavior of Indian adolescents.

## Methodology

This scoping review was conducted to delineate and amalgamate the existing literature on the patterns, trends, and major risk factors among Indian adolescents. This study was conducted according to the Arksey and O’Malley scoping review framework [23] and the Joanna Briggs Institute Reviewers’ Manual [24, 25]. A protocol of this review has already been published [26].

To enhance the quality of the methodology and reporting of the data, this scoping review followed the guidelines described in the PRISMA Extension for Scoping Reviews (PRISMA-ScR) (Supplementary file 1) [27].

### Identifying the research questions

The research questions, search strategy, and eligibility criteria were selected based on the Population, Concept and Context (PCC) strategy [24]. This scoping review aimed to investigate the patterns and trends of adolescent suicide in India and identify the precipitating factors influencing suicidal behaviors among Indian adolescents. To meet this, aim the following research questions were framed:

- What is the evidence available on the patterns and trends of suicide among the adolescent population in India?
- What are the relevant risk factors identified by the evidence affecting adolescent suicide in India?

### Identifying relevant studies:

#### Search strategy

The present review collected relevant literature which was identified from different online databases such as PubMed, SCOPUS, Google Scholar, and PsycINFO. This scoping review literature search was performed by using the various MeSH terms such as, “patterns”; “suicide”; “trends”; “risk factors”; “depression”; “anxiety”, “mental health”; “suicidal tendency”; “suicidal ideation”; “adolescents”; “teenager”; “youth”; and “India” etc. The strategy used for searching the PubMed electronic database is provided in Supplementary file 2. All the grey literature, Communications, case report, and research letter were considered for the review. The reference list of the articles was also searched to find out relevant articles.

### Eligibility criteria

#### Inclusion criteria

- Population: This review considered all studies focusing on suicide among the adolescent group ranging between 10–19 years of age. There was no gender specificity included in the study.
- Concept: For the first objective, various patterns regarding the methods, location, and time of suicide were explored and the trends of the patterns observed were evaluated. And for the second objective, the precipitating influencing factors that act as the risk factors for suicide were studied.
  This review considered studies reporting the patterns, trends, and major risk factors of suicide among Indian adolescents.
- Context: This review considered studies in the Indian context within the study period of 2000 to 2021 and the medium of publication for all the studies was English language only.

#### Exclusion criteria

- Studies undertaken before 2000 and considered populations other than India were excluded.
- Studies targeting individuals below 10 years and above 19 years were excluded.
- Studies conducted other than the English language were excluded.

### Study procedure and selection of the studies

**Fig. 1:**
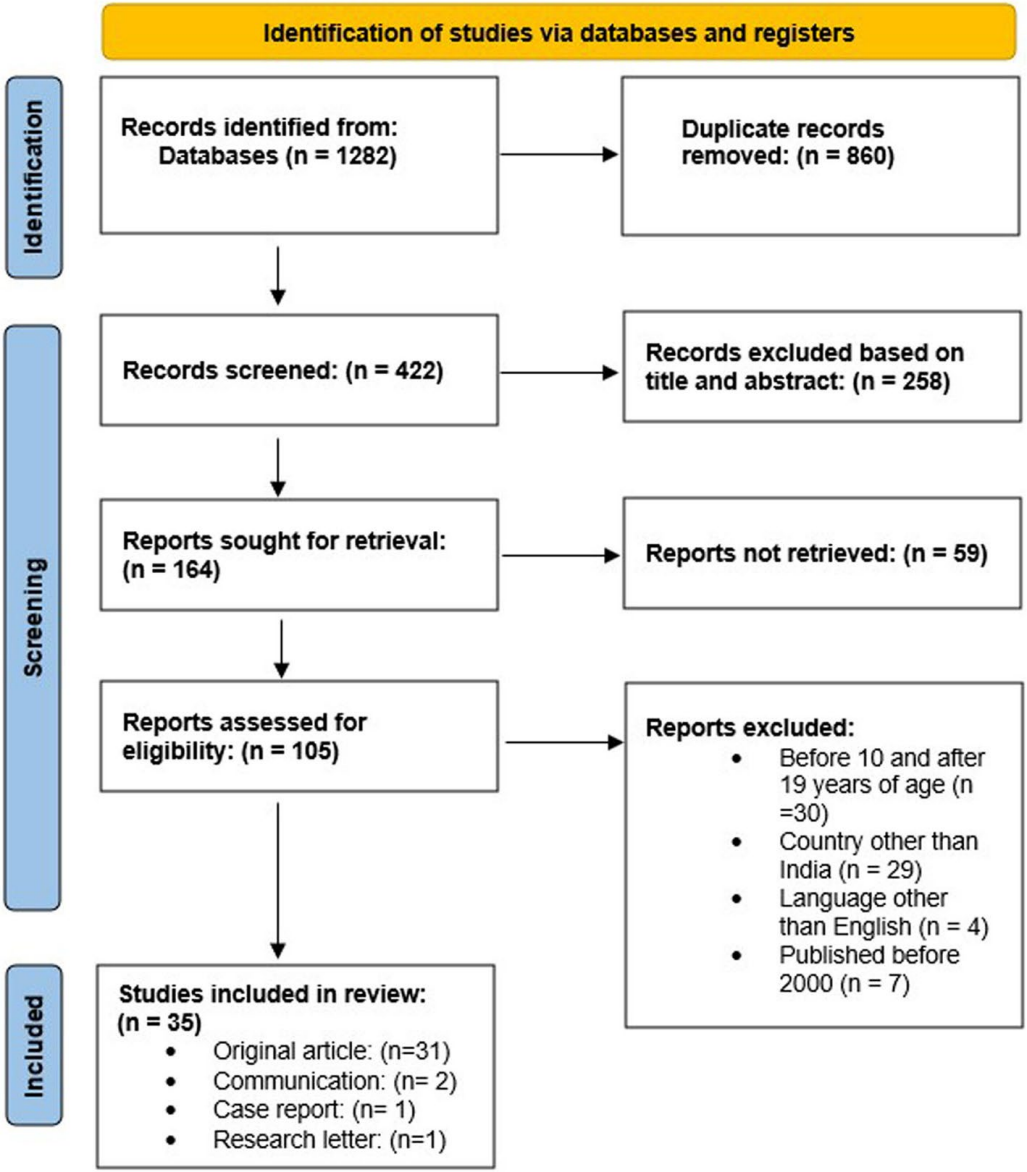
Prisma flow diagram explaining evidence synthesis by detailing the database search and evidence retrieved

To ensure the comprehensiveness of the study, the screening and selection procedure was followed systematically. Titles and abstracts of the original articles were assessed, and duplicate articles were removed from the study by two investigators (RES and SJ) In case of any disagreement, a third reviewer (JP) was consulted. The defined inclusion and exclusion criteria were followed to select the relevant literature. The records identified by the reviewers were included in the full-text screening; the same eligibility criteria were followed to screen the full-text articles. All the stages for the selection of the relevant studies were presented in the flow diagram as prescribed in Preferred Reporting Items for Systemic Review and Meta-analysis Scoping Reviews (PRISMA-ScR) [27] (Fig. 1).

### Charting the data

The reviewers extracted the relevant data manually from the selected full-text articles by the reviewer (RES and SJ) and a predefined data extraction form was developed using a Microsoft Excel spreadsheet for data charting. The key information of the selected studies was extracted based on the research questions. This information includes the title of the paper, year, journal, author, area of study, residency, region, type of study, sample size, age, group, gender, methods, mode of suicide, and risk factors. Before finalization, all the feedback from the investigators was considered to update the data extractions from the included studies.

### Collating, summarizing, and reporting the results

In the final step, the status of existing evidence on patterns, trends, and major risk factors of suicide among Indian adolescents was collected to be categorized into different themes and summarized the results. The retrieved data were coded by identifying the major concepts and themes linked to adolescent suicide. This technique involved labeling sections of data with descriptive codes that reflect the meaning of the text; in the following stage, we arranged similar codes together to uncover broad themes. Once the themes were identified, they were analyzed to better understand the patterns, trends, and major risk factors of suicide among Indian adolescents. The results of the included studies were compiled into a report to identify gaps for further research.

### Ethical considerations

Scoping review does not require ethical approval; it involves a systematic combination and presentation of available resources.

### Patient and public involvement

No patients were involved.

## Results

Through various database searches, we identified 1282 records including grey literature like websites of agencies, academic institutions, and technical bodies. After conducting the deduplication process 422 records were screened where 258 records were excluded based on title and abstract screening. Among the 164 studies sought for retrieval, 59 studies could not be retrieved. Out of the rest 105 records accessed for eligibility, 70 records did not match the inclusion criteria; therefore, a total of 35 records were included in this review containing 31 original research, 2 communications, 1 case report, and 1 research letter.

**Fig. 2:**
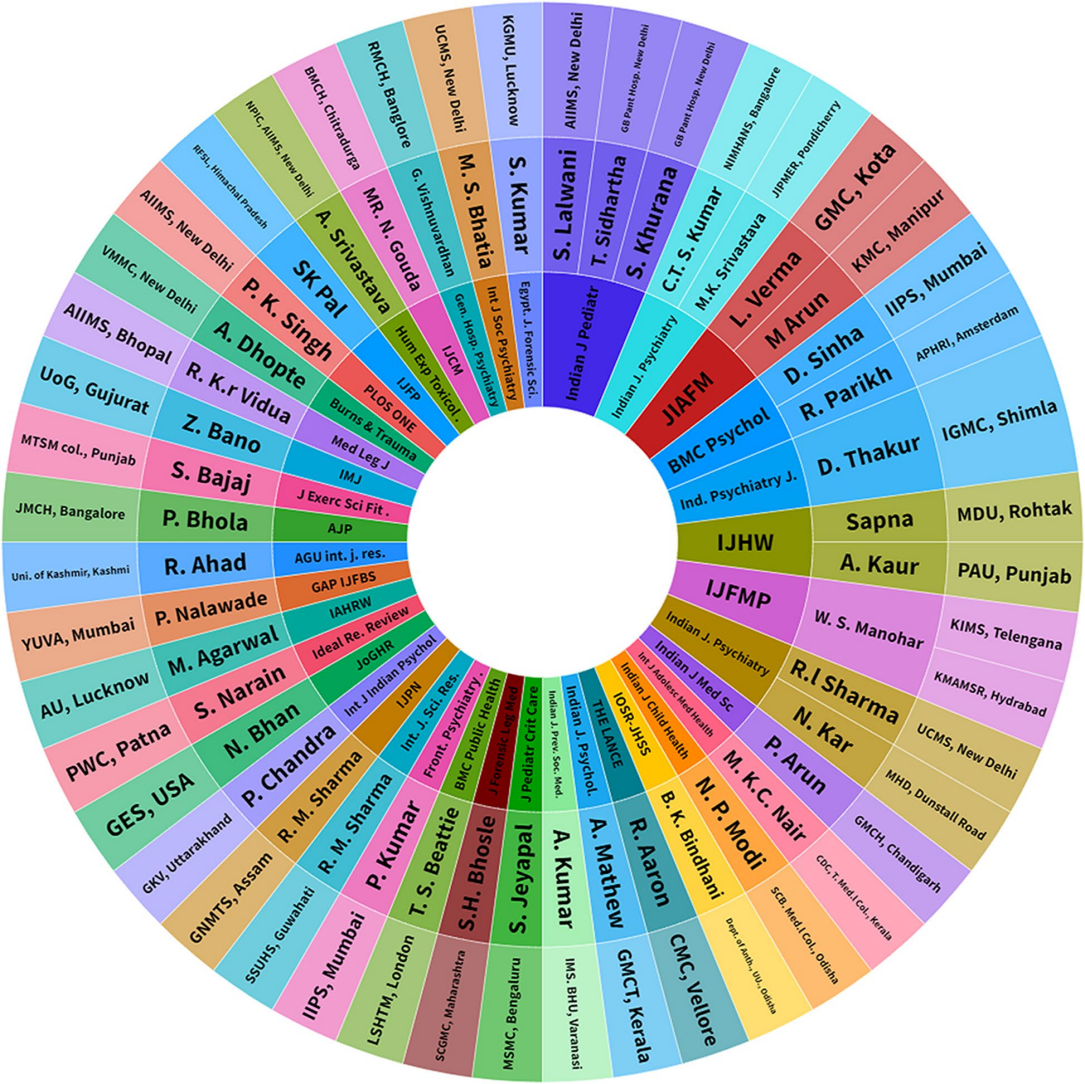
Bibliography of the selected studies presenting pattern of research continuance by researchers, institutional representation, and publishing journals on adolescent suicidal behavior from India

The bibliographic analysis in Fig. 2 shows the overall pattern of research pursued around adolescents’ suicidal behavior by individual researchers in India, institutions involving these studies, and the journals selected for publishing the included studies. The bibliometric analysis indicates that publications are sporadic and there is no consistent pursuit of studies on adolescent suicides in India (Supplementary file 3).

### Characteristics of the included studies

**Fig. 3:**
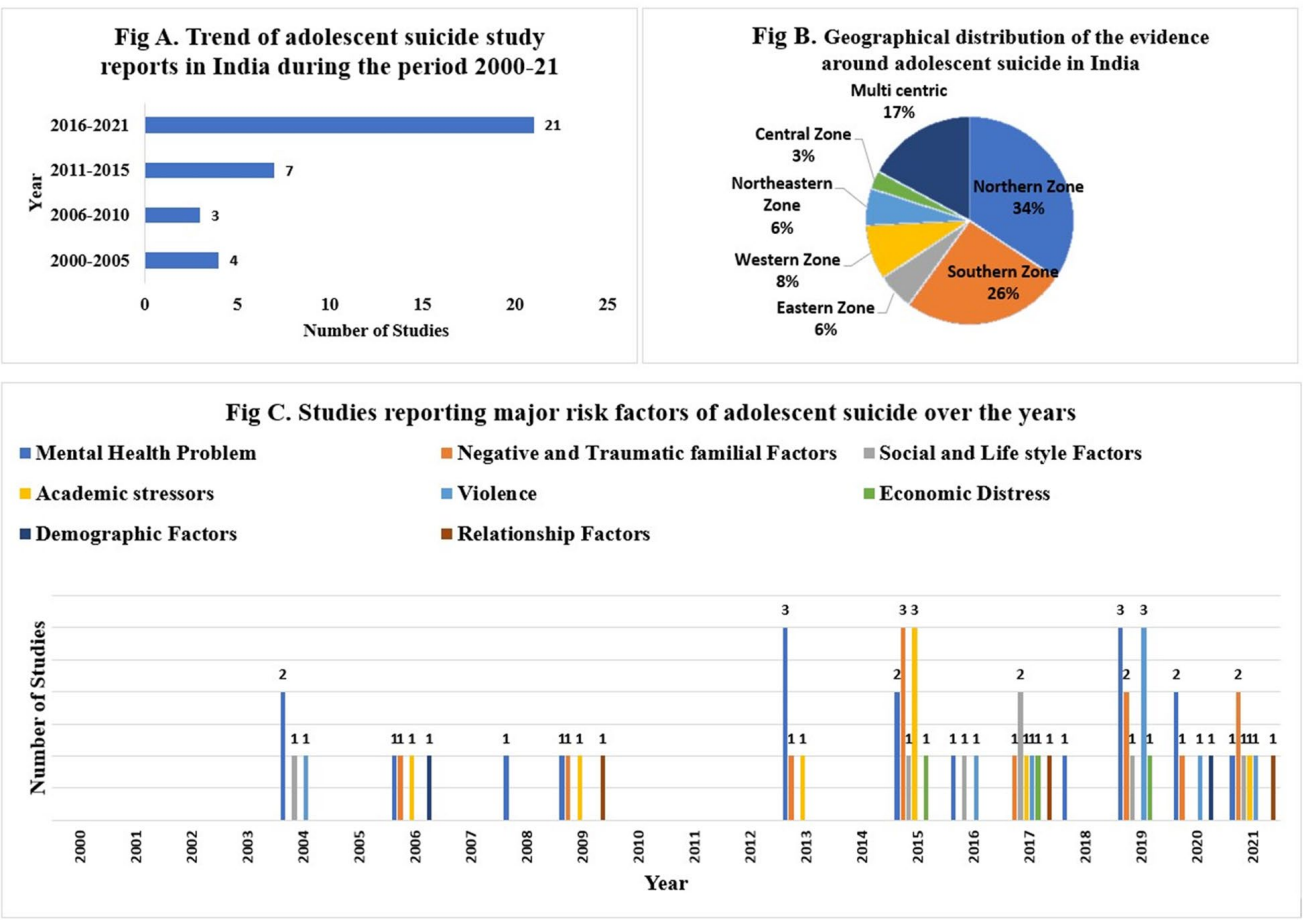
**A** Trend of adolescent suicide study reports in India during the period of 2000–21. **B** Geographical distribution of the evidence around adolescent suicide in India. **C** Studies reporting major risk factors of adolescent suicide over the years

All relevant information such as author, year of publication, studied zone, studied state, studied gender, area of residence, and growth measuring standards for the 35 eligible publications. The number of publications of the included studies showed a fluctuating trend from 2000 to 2021. Among all included studies, only seven papers were reported between the first decade i.e., 2000 to 2010, and most of the eligible studies were published in the last 11 years and post-2010, which eventually demonstrates the rising trends of adolescent suicide in India (Fig. 3A).

### Study population

Sex- stratified analysis was provided in all the studies and 32 of them focused on both men and women. One study focused solely on men, another just on women, and one study was left out. Regarding participants’ age, 14 research studies looked only at people between the ages of 10 and 19 years; however, 18 studies included mixed age groups from which the adolescent age group was taken, and 1 study involving adolescents did not specify the age. The sample size was also retrieved from the selected studies, and it was discovered that 9 studies reported a sample size larger than 1000, while 17 studies reported a sample size greater than 100 but less than 1000 (Table 1).

**Table 1:** Characteristics of the included studies in the present review

SL. NO	Variables of the included studies (*n* = 35)	No. of studies
1	**Type of Article**	
	Original Article	31 (88.57%)
	Communication	2 (5.71%)
	Case report	1 (2.85%)
	Research letter	1 (2.85%)
2	**Type of Study**	
	Quantitative	32 (91.42%)
	Qualitative	2 (5.71%)
	Mixed Method	1 (2.85%)
3	**Type of Sampling techniques**	
	Cross-sectional	32 (91.42%)
	Longitudinal	1 (2.85%)
	Case–control	1 (2.85%)
	Experimental	1 (2.85%)
4	**Place of study**	
	Urban	11 (31.42%)
	Rural	2 (5.71%)
	Both urban and rural	9 (25.71%)
	Not mentioned	13 (37.14%)
5	**Study settings**	
	Community-based	7 (20.00%)
	Hospital-based	10 (28.75%)
	Institution based:	
	• School-based	10 (28.75%)
	• College-based	6 (17.14%)
	• Child observation home	1 (2.85%)
	• Both community and school-based	1 (2.85%)
6	**Studied adolescent group**	
	Total adolescence (10 -19)	5 (14.25%)
	Early adolescence (14 -17)	3 (8.57%)
	Late adolescence (16- 19)	6 (17.14%)
	Mixed age group of adolescence	18 (51.42%)
	N.A	1 (2.85%)
7	**Gender-based sampling type**	
	Male	1 (2.85%)
	Female	1 (2.85%)
	Both male and female	32 (91.42%)
	Not mentioned	1 (2.85%)
8	**Sample size**	
	≤ 100	8 (22.85%)
	≥ 100 and ≤ 1000	17 (48.57%)
	> 1000	9 (25.71%)
9	**Study indicators**	
	Pattern of suicide	10 (28.57%)
	Risk factors of suicide	34 (97.14%)
10	**Studies reporting suicide indicators**	
	Attempted suicide	3 (8.57%)
	Completed suicide	6 (17.14%)
	Deliberate self-harm	3 (8.57%)
	Influenced suicide	1 (2.85%)
	Non-fatal suicidal behaviour (suicidal ideation)	12 (34.28%)
	Suicidal behaviour	4 (11.42%)

### Analysis of the selected studies

Among the included studies various characteristics like the information of the author, year of publication, area of study, residency, region, type of study, sample size, age group, gender, methods, mode of suicide, and risk factors are provided through a summary table (Supplementary file 4). The summary table represented brief information regarding the characteristics of the included studies. After a detailed analysis of the selected studies various patterns, trends, and major risk factors were observed and described below:

## Patterns of suicide among adolescents in India

We found several patterns in the suicide, the season in which the majority of the suicides/attempts were noticed, the time interval as preferred more, the most preferred location for the act/ attempt, and the various methods selected for the suicide.

### Season of suicide

Out of the included studies, two studies discussed the suicide season [28, 29] among Indian adolescents and discovered that the months of March to July had the greatest number of recorded suicides. The trend was explained by the events including the exam results announcements, college admissions, and the start of a new academic session, which frequently take place around this time.

### Time of suicide

The time of suicide among Indian adolescents was recorded in a single study by Manohar et al., 2016 which indicated, the majority of suicides among both male and female adolescents happened during the day rather than at night [30]. Furthermore, the majority of female suicide attempters were either pregnant or menstruating, with those having menstruation cycle-related issues coming in second. These findings indicated that the feeling of physical illness such as dysmenorrhea or abdominal discomfort and hormonal disbalance are the major causes of suicidal behavior of female adolescents.

### Location of committing suicide

Two studies described the location of suicide [28, 31] where it was observed that individuals attempting suicide often selected a familiar location such as their living room or own residence. These findings indicated that the attempters may prioritize easy access to means, and may also be driven by a willingness to avoid being noticed in the event of resuscitation due to the fear of feeling guilty.

### Socio-demographic characteristics of the suicide completers

Among the total included studies, eight studies reported a higher prevalence of suicide among females compared to males [28, 30, 32–37]. Conversely, four studies indicated a higher prevalence among males [36, 38–40]. Nine studies reported that the older adolescent age group faces greater vulnerability to suicide, suicidal ideation, and high suicidal risk behaviour [28, 30, 32, 33, 35, 37–39, 41]. Verma et al., 2021 specifically noted that the venerability to suicide among adolescents increases with age [39]. Additionally, four studies reported that adolescents from low and middle-income families show a higher suicide rate than others [33, 38, 42, 43].

### Means and patterns in reported suicides

From the included studies, 22 studies reported the adopted means of suicide among Indian adolescents. Among all, poisoning and hanging were the two most frequently recorded means of suicide. The next most recorded techniques were suicide by burning, drowning, and falling from a height. There were also reported patterns in the adopted means of suicide among adolescents which we have listed in the following sub-sections.

#### Poisoning

Based on the analysis of the included studies we found that suicide by poisoning, in some studies, was reported with certain patterns like the type of poison used, [28, 39, 43, 44]. Lalwani, 2004 reported that males are more prone to consider poisoning as a mode of suicide than females [28]. We have discussed the detailed patterns of products used for poisoning as reported in included studies in the following sub-sections.

##### Household products

Two studies [33, 42] described household poison used by adolescents. Among these household products, pesticides, phenyl, and kerosene were used for suicide.

##### Agricultural products

One study [42] reported the use of agricultural poisonous products where organophosphorus was used most commonly for committing suicide.

##### Poisonous plants and flowers

Two studies reported about the various poisonous plants/ fruits used by adolescents where Cerbera odollam and Nerium oleander were reported. In the case of flowers yellow oleander was reported [33, 43].

##### Drug overdose

Drug overdose was also a form of poisoning [33, 43] which was reported in two studies. These studies specifically highlighted drug overdose as a cause of poisoning.

#### Hanging

Eight studies reported a variety of patterns in some of the suicide-by-hanging cases, where rope, *chuniri or dupatta* (scarf), saree and soft clothes, iron/guider beams, ceiling fans, and ceiling hooks were the preferred option among adolescents [28–30, 35, 43, 45]. Lalwani, 2004 reported that suicide by hanging was more common among females [28].

#### Burning

Four research studies were thoroughly analyzed, and it was shown that the burning method of suicide was often recorded. where the common method used was by pouring kerosene [28, 30, 33, 43].

#### Drowning

Along with other methods used, suicide by drowning was also noted in the selected three studies [30, 33, 45].

#### Others

There were diverse patterns observed in some of the frequently adopted suicidal methods by adolescents as discussed above. Along with this, a couple of studies reported methods like slashing the wrist [33] and falling from height [28, 32] as frequently adopted modes.

## Trends of adolescent suicide in India

This review comprises 35 studies ranging from the year 2000 to 2021, and after the analysis of these studies, various trends including the number of publications based on the years of publication (Fig. 3A) and the regions of study (Fig. 3B) were observed.

Overall, the findings of the scoping review indicated that the suicide rate among Indian adolescents has been increasing steadily over the past two decades. While there have been some fluctuations in the data, the trend has generally been upward, with the most recent data indicating a sharp increase in the number of suicides among this age group. The studies reported between the first decade ranging from 2000 to 2010 showed no significant trend until 2003 as only one study was reported during these three years of the period; later on, during the years 2004 and 2005, the trend was observed of rising, while this trend took a downward trend until 2010. In the second decade, the year 2013 marked a significant rise in the number of suicide studies among adolescents. From the year 2015 to 2021 a significant number of major studies on adolescent suicides were reported.

### Geographical distribution of the evidence and observed research trends 

From the above-included studies, twelve studies were conducted in the northern region, nine studies in the southern region, six studies were multicentric, two in the eastern region, three in the western region, two in the north-eastern region, and one study in the central region (Table 1). Out of the total studies reported, 11 were studied in urban areas, 2 in rural and 13 were studied in both urban and rural and 13 studies do not mention any study area. When it comes to study setting, the majority of studies were carried out in an institutional setup (*n* = 18/35), followed by hospital-based (*n* = 10/35) and community-based set-ups (*n* = 7/35). **(**Table 1**).**

## Major risk factors of suicide in the reported studies

Out of all the included articles, 35 studies reported on the major risk factors for adolescent suicide. Nineteen studies reported mental health problems as the most occurrent risk factor while negative and traumatic familial factors were reported in 12 studies. Additionally, social and lifestyle factors were reported in 7 studies, academic stressors in 8 studies, violence in 8 studies, economic distress in 3 studies, relationship factors in 3 studies, and demography (age, gender, and lower economic status) in 3 studies (Fig. 3C).

Figure 3C represents the year-wise publication of the major risk factors among adolescents. It was observed that in the first decade and early second decade i.e., from the year 2000 through 2010 to 2012, research on the major risk factors was limited. In the years 2000, 2001,2002,2003,2005, 2007, 2010, 2011, 2012, and 2014 no study reported the risk factors. This indicates that the focus of exploring major risk factors of suicide in India is recent and is less explored. During the second decade i.e., from 2013 to 2021, the studies have reported the importance of studying the risk factors and hence the number of studies on the major risk factor has increased within that period

### Mental health problems

Among the included studies, 19 studies reported mental health problems as a concern and reason for suicide of which personality disorder and mental illness were reported in 3 studies [28, 33, 45]. while suffering from conduct disorder with alcohol abuse, alcohol dependence syndrome, and psychoses were reported in two studies [28, 33].

Among the other mental health problems depression was found as the most occurrent risk factor [28, 37, 38, 43, 46–48] followed by suicidal ideation was reported in 12 studies [32, 36, 37, 40, 47, 49–55] Eight studies [28, 29, 32, 33, 35, 47, 56, 57] observed stress, four studies [28, 38, 53, 58, 28, 55] described suicide due to anxiety, and one research [28] indicated helplessness as a contributing factor.

### Negative and traumatic familial issues

A significant number of studies (*n* = 12) reported on the children-parent relationships where the findings showed that adolescents doing suicide had a history of either feeling neglected by their parents [32], or were highly ignored [35]; some children had a history of adversely being affected by the constant criticism and highly restrictive behavior of parents [29], while disagreement between parents and children [29] and deviant parenting [48] were other major factors driving the children to the extreme step of suicide. Family history of mental illness [41, 59] and substance abuse [41, 59] was other major influencing factor reported in the selected studies. Conflicts in the family were found as the third most negatively impacting factor for children leading to suicide in the reported studies [33, 34, 42]. High expectations by parents and teachers [29, 35], loss of near and dears [29, 33, 34](viz. the death of a family member and death of a puppy) [33], disturbed relationships among the family members [59], and the changes in family obligations and decision making, as well as modifications in the child-rearing and socialization processes, were found the other most occurring risk factors for self-harm among children. Other risk factors like the frequent argument among parents [29], physical abuse by parents [32], sexual abuse to the mother [31], and being a single child [48] were also found risks for suicidal tendencies as reported in a study.

### Social and lifestyle factors

The association between social life and suicide among adolescents was reported as a major factor in seven studies. The findings showed that substance abuse was noted in three studies as a major factor leading to suicide [34, 42, 46], followed by addiction to the internet [30, 45, 46]. lack of friends circle/ social life and poor interpersonal relationships resulted in more of a mechanical life among the adolescents which increased psychological distress by further resulting in suicidal tendencies and such attempts [30]. Shaming and discriminatory behavior in the peer group premarital pregnancy were also noticed as factors of suicide in the selected studies Suicide due to the Blue Whale game was also reported in one study [56].

### Academic stressors

Eight studies reported the academic stressors among adolescents where school-related issues [32, 37, 42, 48], failure in examination [33, 34], the decline in academic performance [29], lack of clarity about academic future [29], and poor class performance [32] were reported as factors pushing children’s suicide attempts.

### Violence

Eight studies examined the association between violence (both physical and psycho-social) and adolescent suicide in India. Under the risk factors, the most occurrent risk factor was domestic quarrel [30, 34, 45] reported in three studies, bullying [3] in two studies, involvement in fights [46], aggression [60], sexual assault, and rape [57] and parental marital violence and personal experience of marital violence [36] were found in major reported factors pushing the children towards suicide.

### Economic distress

Among the included studies three studies describe the relationship between economic distress and suicide, where financial crisis [34, 41] was reported in two papers and unemployment was reported in a single study [37].

### Relationship factors

The association between failed personal relationships and suicide was reported in three papers. While failure in love was found the reason in one study [34], unreciprocated love [29] and dislike by peers [29] were also found as other risk factors of suicide. Verma et al., (2021) reported that female adolescents faced more problems like sexual assault, love failure, unwanted marriage, unwanted pregnancies, and less emotional support from family members leading to self-harm attitudes and increased suicidal deaths [39].

## Discussion

The present scoping review aimed to investigate scientific literature reporting suicide among Indian adolescents and to summate the understanding of the patterns in the methods of suicide; it also explored the precipitating risk factors influencing suicidal behavior. We also studied the trend of research in adolescent suicide in India between the years 2000 to 2021 for a broader outlook of adolescent suicide at the national level.

Over the past 20 years, the trend of suicide among Indian adolescents showed a rising trend. As per international data, suicide incidence increased more among women than men [4]. The increase in suicide rates among females could be attributed to several factors, including gender-specific stressors, such as gender-based violence, discrimination, and social norms that place a higher burden on females [61]. The rise in male suicide rates could be attributed to several variables, such as financial strain, academic pressure, and substance abuse [62]. Increased research priorities around mental health in the last decade indicated the excessive use of the internet is a major concern among adolescents as it is leading them to dangerous and addictive internet-related activities including gaming [63]. Modernity-driven drastic changes in family structure, social kinship, and social life are leading to increasing use of social media among adolescents causing major harm. Our analysis also found the presence of most of the adolescent suicide-related risk factors within this last decade, which might be due to an increase in the number of studies.

The present scoping review identified several patterns associated with adolescent suicide in India. For example, the period from March to July was found as a high suicide reporting period which might have the root in the publication of school examination results. Such suicides were also observed of happening during the daytime in comparison to night, a known location in compared to any unacquainted place for better exaction and overcoming attention. [28]. Among the methods used for suicide among adolescents, the most common method was poisoning followed by hanging, which is consistent with global trends [4]. Suicide was more common among rural adolescents than their urban counterparts which could be attributed to a variety of variables such as restricted access to mental health services, social isolation, and economic stress [64]. Similarly, suicide was also reported as more prevalent among adolescents from low socioeconomic backgrounds as well as less-educated families [42, 56].

In our study, we found that the major risk factors leading to suicide or such attempts are mental health problems, negative and traumatic familial factors, social and lifestyle factors, academic stressors, violence (physical and psycho-social), economic distress, relationship factors, female gender, and late adolescence. Those having previous sexual abuse episodes dropped out of school and poor parental emotional support were observed as at higher risk of suicidal ideation and attempts [47].

It is important to note that while conducting our literature review, we came across additional 14 studies reporting suicide among children and adolescents in India, as a result of the inadequately defined age groups of the participants in 14 publications, so we had to exclude those papers from our analysis. It is crucial to highlight, that these studies' findings remain significant and useful for a better understanding of mental health among Indian adolescents. Psychiatric traits like depression, anxiety, and poor self-esteem were the major factors that led to suicide as reported in many studies [14, 47, 65–67]. In a Case report by Vidua (2020), it was found that a 20-year-old boy committed suicide when his parents refuse to recharge his phone so he could play PUBG [68]. A study by Srivastava (2005) found that adolescents (12–18 years) intentionally attempt reported in 20% of the total sample [65]. According to a study by Kar (2010), childhood trauma contributes to adult (20–29 years old) suicide attempts [69]. A study by Gauda (2008) reported that School related issue was an important factor that leads to suicide among youngsters [70]. Moreover, to have a more comprehensive understanding of the issue and to identify solutions, these studies need to be looked at.

In the above context, we observed that the available evidence is incomprehensive and limited in finding while researches are inconsistent. By analyzing the authors/ corresponding authors, affiliated institutions, and the publishing journals, we observed no such consistency from the reporting research groups or scientists, and institutes in pursuing studies on adolescent suicidal behavior. Rather the studies are more incidental and sporadic (Fig. 2). This circumstance highlights the critical need for more consistent and focused research on India's adolescent mental health. Addressing these research gaps will result in new, more inclusive, and complete research outputs in adolescent health, leading to the establishment of efficient policies for adolescents.

**Fig. 4:**
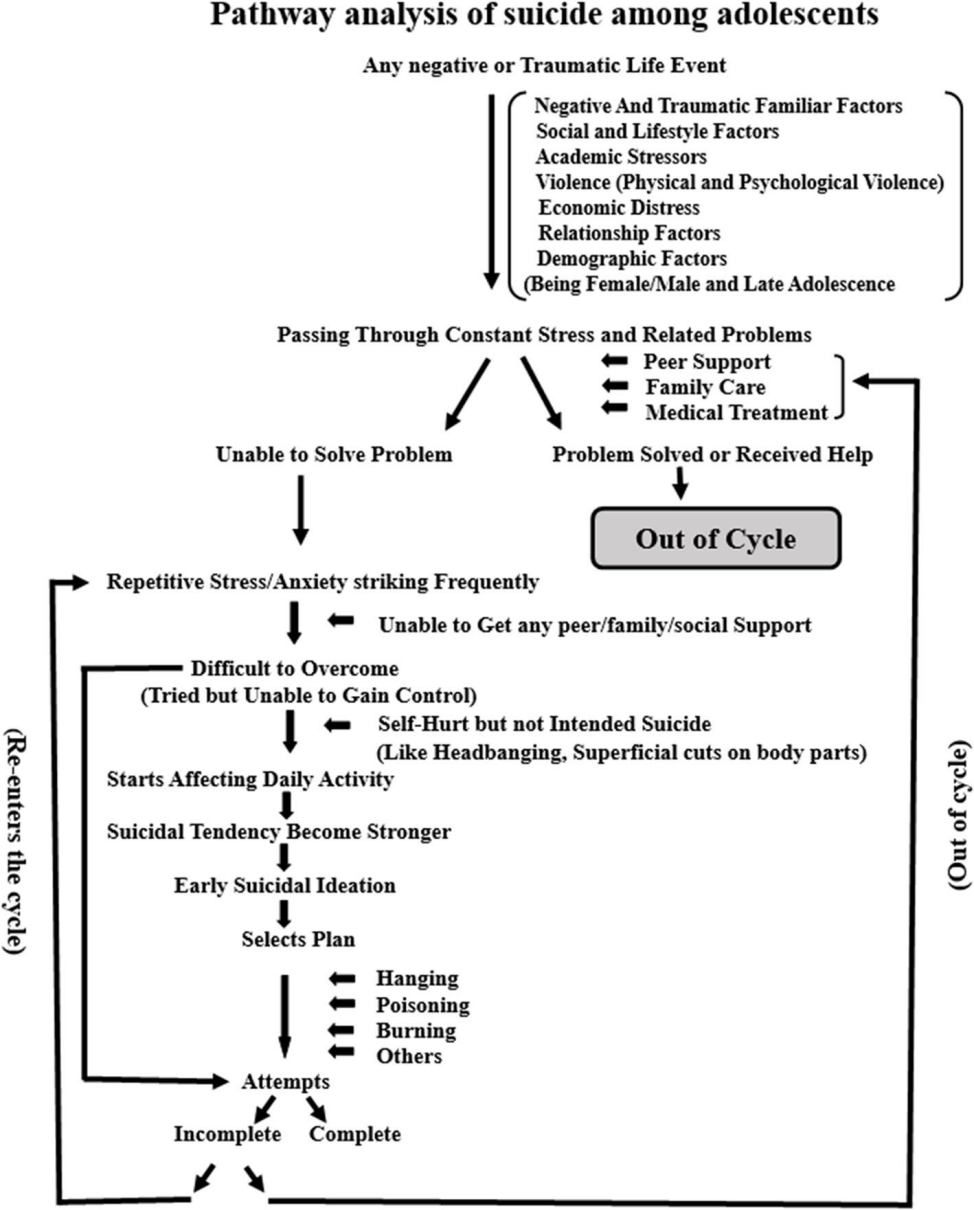
A pathway analysis of suicide attempts among adolescents

In the present analysis, we also have tried to explain the possible pathway of suicide attempts among adolescents (Fig. 4) based on the findings of the selected studies. A series of continuous and interdependent factors were observed that were together forming a loop or in other words, a psychological cycle leading to poor mental health [71]. It was observed that any negative or traumatic life events can become the triggering factors pushing the individuals into this loop. Intensity than the extensity of the negative events was observed of becoming the leading causes of suicide even in smaller happenings [72]. When the individual finds it extremely difficult to escape the negativity or failure, he/she starts idealizing suicide as the last resort for solving or coming out of the problem [73]. The increase in the rate of suicidal ideation is seen due to the inability to cope with the additional problems that life throws during a bad mental state [74]. This eventually leads to premature suicidal ideation and the means of suicide is perceived as the easily available option to the individual. This is followed by events when s/he decides to make an attempt by selecting a plan [75]. The attempt may or may not result in death. However, if s/he succeeds to overcome the problem and receives proper care and support, s/he may come out of the cycle and can be saved [76]. This cycle demonstrates the importance of peer and family support for suicide prevention in the changing and rapidly evolving social structure, the role of family, peer group, and kin is evolving as vital for better mental health and suicide prevention [77].

Under the Indian penal code 309, suicide remained a criminal act as well as a non-cognizable offense until the year 2018. With Mental Health Act 2017 which was passed in the parliament in 2019, suicide was decriminalized in India [78]. However, mental health still remains a strong stigma in the Indian population due to various attached social, cultural, and moral values. All these scenarios are creating negative attitudes toward seeking help for mental health issues like anxiety, depression, etc. which makes it very difficult for a person to prevent suicide [79].

## Gaps analysis

While conducting the present review, we found several gaps in the suicide studies in India. From the methodological point of view, most of the studies were cross-sectional and epidemiological design reporting quantitative data while two studies were vaguely defined as experimental and case–control; one study was a secondary retrospective design. We did not find any qualitative or longitudinal or cohort studies, which helps to give an in-depth understanding of the root cause of the problem. Out of 35 studies, the study settings like urban/rural/ tribal/slum have not been defined in 13 studies while in another 7, settings-wise samples have not been clarified. Similarly, population-wise, there is no study on indigenous/tribal and vulnerable populations like slums. About 80% of studies are institution-based (school, college or hospital, etc.). Age group-wise, only about 15% (5) of studies covered the whole 10–19 years. In the context of adolescent gender, only male and female adolescents were studied, and no study considered suicide among the third gender. Fifteen of the thirty-five studies have defined the sample design (method and sample size) properly. The present study also found 19 studies using various mental health scales however have either not validated or not explained the status of validation in the paper.

Studies among high-risk individuals (like among individuals with a history of self-harm, abuse, juveniles in jail, etc.) are also limited in number and scope; hence in the overall scenario of research on adolescent suicide, the suicide associate risk factors are understood poorly or sporadically leading to limited knowledge towards scopes of prevention in India in the local and cultural context. Additionally, suicide in public places and live on social media sites were not exclusively reported in the studies. It was also noted that little research was done on the protective factors that could help adolescents to prevent idealizing suicide as a last resort for their problems. Studies on roles played by media through reporting (either print or electronic) have not also been explored adequately. Though there are several particular communities with a high risk of suicidal behavior in India, studies are less in such directions.

While the government attempts to enhance mental healthcare through programs such as the Rashtriya Bal Swasthya Karyakram (RBSK), there is currently a lack of adequate studies evaluating the effectiveness of such programs to decrease suicide among adolescents. Furthermore, India's Mental Health Act of 2017, which is meant to protect the rights of individuals dealing with mental illnesses, has not been fully implemented. however, school health programs have been started to address mental health concerns in schools, and more studies are required to understand their effects. In addition, media reports on suicide are presented in an inappropriate manner which influences more youngsters to commit suicide. In India, addressing adolescent suicide represents a major challenge because of the stigma connected with mental health and how people in the community determine and indicate mental illness.

## Strengths and limitations of the study

This scoping review is the first of such studies on the topic of the patterns, trends and major risk factors of suicide among Indian adolescents. This evidence-based study will be highly beneficial for further research and future research policy. This study is limited to the English language and electronically available databases. In the course of this scoping review, non-retrieval of any additional evidence may mainly be attributed to limited access to databases, restriction of literature search to English language only, limited availability of data on specific selected aspects, variations in terminologies used across studies etc.

## Conclusion

Suicide among Indian adolescents has become a significant concern in the recent years. Addressing the issue of Adolescent suicide requires a holistic approach. The finding indicates that various factors influence adolescent suicide, including psychiatric, sociocultural, individual, and environmental factors. Preventing adolescent suicide need a multi-faceted approach including, increasing awareness, reducing the stigma associated with mental health, implementing early ideation and intervention, strengthening the adolescent support system, and, improving access to mental health services. Suicide prevention involves strong collaborative cooperation between various stakeholders, including policymakers, teachers, parents, and adolescents themselves. It is crucial to prioritize adolescents' mental health needs and give them the resources and support networks they require to overcome their problems. Future research should be focused on generating more longitudinal studies, in-depth qualitative investigations, and evaluating the impact of prevention programs to enhance the research and strengthen prevention policies and interventions.

## Recommendations

### Research recommendations

From a research perspective, it is imperative to enhance the scientific rigor of studies by ensuring a robust methodology. Authors need to ensure the availability of explicit details on the study and sample design, sampling strategy, and application of tools for data collection. Research efforts should transcend prevalence description studies and explore socio-behavioral pathways of suicide, considering diverse cultural contexts. Implementation research in mental health for the control and prevention of suicide in India is crucial, necessitating longitudinal studies better for a comprehensive understanding of the adolescent behavioral aberration. Evaluations of the scope of healthy reporting of suicide in media should be prioritized.

### Policy and programmatic recommendations

The existing national and state-level programs like the Rashtriya Kishor Swastya Karyakram (RKSK), and Ayushman Bharat have much more scope to deliver in program implementation level. Improved implementation policy and periodic assessments of ongoing practices towards emerging and continuing adolescent mental health issues hold the key. In the highly diverse Indian society, the policies need to accommodate various social and cultural factors, including ethnicity, locality, social status, various economic strata, and beliefs to effectively reach every adolescent in need.

Furthermore, addressing socio-cultural stigma and negative attitudes towards mental health and associated health-seeking behavior still stands as the biggest challenge. Long-term, consistent, and culturally appropriate measures are essential for overcoming these barriers.

### Promoting protective factors

Promotive and Protective factors play a crucial role in reducing suicidal behavior [80]. Efforts should focus on promoting positive thoughts and activities, emphasizing the importance of meaningful life, proper diet, increasing the chances of healthy communication in child-parent relationships, practicing yoga and meditation, and journaling the triggering thoughts could act as a protective factor [81]. In the context of suicide prevention, various innovative interventions could be developed in India like the gatekeeper technique [82], school-based interventions, parent-children interface, etc. should be explored for rising awareness on dealing with poor mental health issues.

### School-based interventions

Regular programs that focus on mental health awareness, stress management, and counselling services within all educational institutions and particularly government settings require prioritisation. These initiatives aim to create a supportive and nurturing environment that contributes to the overall well-being of students.

### Parent-children interface

This suggests the need for interventions that facilitate open communication and understanding between parents and their adolescent children regarding mental health issues. Strengthening the parent–child relationship, fostering effective communication, and details to the parents to recognize and respond to signs of mental health concerns in their children are crucial aspects of this recommendation.

### Adaptation of country-specific strategies

In addition to the above, we acknowledge the importance of implementing and scaling up of already available country-specific suicide prevention and crisis support services such as TeleMANAS (National Tele Mental Health Programme of India, 2023) [83].

In spite of all the above efforts, we are aware of the fact that only the tip of the iceberg of poor mental health challenges faced by adolescents has been explored. Hence, extensive, consistent, and innovative research in this direction remains pivotal.

### Supplementary Information

**Additional file 1.** Preferred Reporting Items for Systematic reviews and Meta-Analyses extension for Scoping Reviews (PRISMA-ScR) Checklist. 

**Additional file 2.** Search strategy MEDLINE (PubMed).

**Additional file 3.** List of journals and Institutes of 1^st^ Authors.

**Additional file 4.** Data extraction table.

## Data Availability

The data used for analysis is available within the paper and supplementary file.

## Abbreviations

NCRB: National Crime Records Bureau
PCC: Population, concept and context strategy
PRISMA-Scr: Preferred reporting items for systemic review and meta-analysis scoping reviews
MeSH: Medical subject headings
PUBG: Players unknown battle ground
RKSK: Rashtriya Kishor Swastya Karyakram
