# Supplementary material for: The patterns, trends and major risk factors of suicide among Indian adolescents – a scoping review
Source: BMC Psychiatry. 2024 Jan 9;24:35. doi: 10.1186/s12888-023-05447-8 (PMC10775453; doi:10.1186/s12888-023-05447-8)
Supplement: Supplementary file 2 — Additional file 2. Search strategy MEDLINE (PubMed). [file 12888_2023_5447_MOESM2_ESM.docx]

**Supplementary file 2**

**Search strategy MEDLINE (PubMed)**

Search conducted Aug 20, 2022, date range from January 2000 to December 2021

| Search | Query | Records retrieved |
| --- | --- | --- |
| #1 | "suicid"[All Fields] OR "suicidal ideation"[MeSH Terms] OR ("suicidal"[All Fields] AND "ideation"[All Fields]) OR "suicidal ideation"[All Fields] OR "suicidality"[All Fields] OR "suicidal"[All Fields] OR "suicidally"[All Fields] OR "suicidals"[All Fields] OR "suicide"[MeSH Terms] OR "suicide"[All Fields] OR "suicides"[All Fields] OR "suicide s"[All Fields] OR "suicided"[All Fields] OR "suiciders"[All Fields] | 113,458 |
| #2 | ("suicid"[All Fields] OR "suicidal ideation"[MeSH Terms] OR ("suicidal"[All Fields] AND "ideation"[All Fields]) OR "suicidal ideation"[All Fields] OR "suicidality"[All Fields] OR "suicidal"[All Fields] OR "suicidally"[All Fields] OR "suicidals"[All Fields] OR "suicide"[MeSH Terms] OR "suicide"[All Fields] OR "suicides"[All Fields] OR "suicide s"[All Fields] OR "suicided"[All Fields] OR "suiciders"[All Fields]) AND ("behavior"[MeSH Terms] OR "behavior"[All Fields] OR "behavioral"[All Fields] OR "behavioural"[All Fields] OR "behavior s"[All Fields] OR "behaviorally"[All Fields] OR "behaviour"[All Fields] OR "behaviourally"[All Fields] OR "behaviours"[All Fields] OR "behaviors"[All Fields] OR "pattern"[All Fields] OR "pattern s"[All Fields] OR "patternability"[All Fields] OR "patternable"[All Fields] OR "patterned"[All Fields] OR "patterning"[All Fields] OR "patternings"[All Fields] OR "patterns"[All Fields]) AND ("trend"[All Fields] OR "trended"[All Fields] OR "trending"[All Fields] OR "trends"[MeSH Subheading] OR "trends"[All Fields]) AND ("risk"[MeSH Terms] OR "risk"[All Fields]) AND ("suicidal ideation"[MeSH Terms] OR ("suicidal"[All Fields] AND "ideation"[All Fields]) OR "suicidal ideation"[All Fields]) | 457 |
| #3 | "depressed"[All Fields] OR "depression"[MeSH Terms] OR "depression"[All Fields] OR "depressions"[All Fields] OR "depression s"[All Fields] OR "depressive disorder"[MeSH Terms] OR ("depressive"[All Fields] AND "disorder"[All Fields]) OR "depressive disorder"[All Fields] OR "depressivity"[All Fields] OR "depressive"[All Fields] OR "depressively"[All Fields] OR "depressiveness"[All Fields] OR "depressives"[All Fields] | 600,711 |
| #4 | "mental health"[MeSH Terms] OR ("mental"[All Fields] AND "health"[All Fields]) OR "mental health"[All Fields] | 491,619 |
| #5 | ("adolescences"[All Fields] OR "adolescency"[All Fields] OR "adolescent"[MeSH Terms] OR "adolescent"[All Fields] OR "adolescence"[All Fields] OR "adolescents"[All Fields] OR "adolescent s"[All Fields]) AND ("adolescent"[MeSH Terms] OR "adolescent"[All Fields] OR "youth"[All Fields] OR "youths"[All Fields] OR "youth s"[All Fields]) AND ("adolescent"[MeSH Terms] OR "adolescent"[All Fields] OR "teenage"[All Fields] OR "teenager"[All Fields] OR "teenagers"[All Fields] OR "teenaged"[All Fields] OR "teenager s"[All Fields] OR "teenages"[All Fields]) | 2,277,111 |
| #6 | "india"[MeSH Terms] OR "india"[All Fields] OR "india s"[All Fields] OR "indias"[All Fields] | 743,105 |
| #7 | #1 AND #2 AND #3 AND #4 AND #5 AND #6 | 868 |
