# Supplementary material for: The patterns, trends and major risk factors of suicide among Indian adolescents – a scoping review
Source: BMC Psychiatry. 2024 Jan 9;24:35. doi: 10.1186/s12888-023-05447-8 (PMC10775453; doi:10.1186/s12888-023-05447-8)
Supplement: Supplementary file 3 — Additional file 3. List of journals and Institutes of 1st Authors. [file 12888_2023_5447_MOESM3_ESM.docx]

**Supplementary File 3**:

List of journals and Institutes of 1^st^ Authors.

| **Sl no.** | **Authors Name** | **Institute Name** | **Institute Abbreviation** | **Journal Name** | **Journal Abbreviation** |
| --- | --- | --- | --- | --- | --- |
| 1 | S. Lalwani | All India Institute of Medical Sciences, New Delhi | AIIMS, New Delhi | Indian Journal of Pediatrics | Indian J Pediatr |
| 2 | T. Sidhartha | G. B. Pant Hospital, New Delhi | GB Pant Hosp. New Delhi | Indian Journal of Pediatrics | Indian J Pediatr |
| 3 | P. Arun | Government Medical College and Hospital, Chandigarh, India | GMCH, Chandigarh | Indian J Med Sc | Indian J Med Sc |
| 4 | M. K.C. Nair | Child Development Center , Thiruvananthapuram Medical College, Kerala , India | CDC, T. Med.l Col., Kerala | Int J Adolesc Med Health | Int J Adolesc Med Health |
| 5 | N. P. Modi | SCB Medical College, Cuttack, Odisha | SCB. Med.l Col., Odisha | Indian J Child Health | Indian J Child Health |
| 6 | B. K. Bindhani | Department of Anthropology, Centre for Advances Studies, Utkal University | Dept. of Anth., UU., Odisha | IOSR Journal Of Humanities And Social Science | IOSR-JHSS |
| 7 | R. Aaron | Christian Medical College, Vellore | CMC, Vellore | THE LANCE | THE LANCE |
| 8 | A. Mathew | Government Medical College, Trivandrum, Kerala | GMCT, Kerala | Indian Journal of Psychological Medicine | Indian J. Psychol. |
| 9 | C.T. S. Kumar | Department of Psychiarty, NIMHANS, Bangalore | NIMHANS, Bangalore | Indian Journal of Psychiatry | Indian J. Psychiatry |
| 10 | A. Kumar | Institute of Medical Sciences, BHU, Varanasi | IMS. BHU, Varanasi | Indian J. Prev. Soc. Med. | Indian J. Prev. Soc. Med. |
| 11 | S. Jeyapal | Mazumdar Shaw Medical Centre, Paediatric Intensive Care Unit, Narayana Hrudayalaya, Bengaluru, Karnatak | MSMC, Bengaluru | Journal of Pediatric Critical Care | J Pediatr Crit Care |
| 12 | L. Verma | Govt Medical College, Kota, Rajasthan | GMC, Kota | J Indian Acad Forensic Med. | JIAFM |
| 13 | S.H. Bhosle | Shankarrao Chavan Government Medical College, Nanded, Maharashtra | SCGMC, Maharashtra | Journal of Forensic and Legal Medicine | J Forensic Leg Med |
| 14 | D. Sinha | International Institute for Population Sciences, Mumbai, Maharashtra | IIPS, Mumbai | BMC Psychology | BMC Psychol |
| 15 | T. S. Beattie | London School of Hygiene and Tropical Medicine, London | LSHTM, London | BMC Public Health | BMC Public Health |
| 16 | P. Kumar | International Institute for Population Sciences (IIPS), Mumbai | IIPS, Mumbai | Frontiers in Psychiatry | Front. Psychiatry . |
| 17 | R. M. Sharma | Srimanta Sankardeva University of Health Sciences, Guwahati, Assam | SSUHS, Guwahati | International Journal of Scientific and Research Publications | Int. J. Sci. Res. |
| 18 | R. M. Sharma | GNM Training School, Goalpara, Assam | GNMTS, Assam | Indian Journal of Psychiatric Nursing \| | IJPN |
| 19 | P. Chandra | Gurukula Kangri Vishwavidyalaya, Haridwar, Uttarakhand | GKV, Uttarakhand | The International Journal of Indian Psychology | Int J Indian Psychol |
| 20 | N. Bhan | Center on Gender Equity and Health, University of California, San Diego, California, USA | GES, USA | Journal of global health repprts | JoGHR |
| 21 | S. Narain | Department of Psychology, Patna Women’s College (Autonomous) Patna University | PWC, Patna | Ideal Research Review | Ideal Re. Review |
| 22 | S. Khurana | G.B. Pant Hospital, New Delhi | GB Pant Hosp. New Delhi | Indian Journal of Pediatrics, | Indian J Pediatr |
| 23 | D. Thakur | Indira Gandhi Medical College, Shimla, | IGMC, Simla | Industrial Psychiatry Journal | Ind. Psychiatry J. |
| 24 | Sapna | Maharishi Dayanand University, Rohtak | MDU, Rohtak | Indian Journal of Health and Wellbeing | IJHW |
| 25 | W. S. Manohar | Kamineni Institute of Medical Sciences, Narketpally, District Nalgonda, State Telangana. | KIMS, Telengana | Indian Journal of Forensic Medicine and Pathology | IJFMP |
| 26 | M. Agarwal | Amity University Uttar Pradesh, Lucknow | AU, Lucknow | Indian Association of Health, Research and Welfare | IAHRW |
| 27 | P. Nalawade | Youth for Unity and Voluntary Action (YUVA), Mumbai, Maharashtra | YUVA, Mumbai | GAP INDIAN JOURNAL OF FORENSICS AND BEHAVIOURAL SCIENCES | GAP IJFBS |
| 28 | R. Parikh | Amsterdam Public Health research institute, Vrije Universiteit Amsterdam | APHRI, Amsterdam | BMC Psychology | BMC Psychol |
| 29 | W. S. Manohar | Kamineni Academy of Medical Sciences and Research Center, L. B. Nagar, Hyderabad | KMAMSR, Hydrabad | Indian Journal of Forensic Medicine and Pathology | IJFMP |
| 30 | R. Ahad | University of Kashmir | Uni. of Kashmir, Kashmi | AGU International Journal of Research in Social Sciences & Humanities | AGU int. j. res. |
| 31 | P. Bhola | St. John’s Medical College & Hospital, Bangalore | JMCH, Bangalore | Asian Journal of Psychiatry | AJP |
| 32 | S. Bajaj | Master Tara Singh Memorial College for Women Ludhiana (Punjab) India | MTSM col., Punjab | Journal of Exercise Science & Physiotherapy | J Exerc Sci Fit . |
| 33 | A. Kaur | Punjab Agricultural University, Ludhiana, Punjab | PAU, Punjab | Indian Journal of Health and Well-being | IJHW |
| 34 | Z. Bano | University of Gujrat | UoG, Gujurat | Isra Med J. | IMJ |
| 35 | R.l Sharma | University College of Medical Sciences, New Delhi | UCMS, New Delhi | Indian J Psychiatry | Indian J. Psychiatry |
