# Supplementary material for: The patterns, trends and major risk factors of suicide among Indian adolescents – a scoping review
Source: BMC Psychiatry. 2024 Jan 9;24:35. doi: 10.1186/s12888-023-05447-8 (PMC10775453; doi:10.1186/s12888-023-05447-8)
Supplement: Supplementary file 4 — Additional file 4. Data extraction table. [file 12888_2023_5447_MOESM4_ESM.docx]

**Supplementary file 4**

**Data Extraction Table:**

| **SL NO.** | **AUTHOR (YEAR)** | **AREA OF STUDY; STUDY DESIGN** | **STUDY TYPE** | **RESID-ENCY** | **AGE GROUP; SAMPLE SIZE** | **GENDER** | **SETTING** | **SUICIDAL BEHAVIOUR** | **MODE OF SUICIDE ( PATTERN)** | **RISK FACTORS** |
| --- | --- | --- | --- | --- | --- | --- | --- | --- | --- | --- |
| 1 | Lalwani et.al (2004) | South Delhi ; cross-sectional | quantitative | Urban | 10 to 18; 222 | both male and female | Hospital-based | completed suicide | hanging,  poisoning,  burns, fall from height | depression, anxiety |
| 2 | Sidharta et.al  (2006) | Central Delhi; cross-sectional | quantitative | Urban | 12 to 19; 1205 | both male and female | School-based | NFSB and deliberate self-harm | poisoning, overdose of medicine,  cutting self,  hanging,  jumping | Demographic risk factors, Family environment, School environment, death wish and deliberate sself-harm |
| 3 | Arun et.al (2009) | Chandigarh; cross-sectional | quantitative | Urban | students from class 7 to 12; 2402 | both male and female | School-based | suicidal ideas | n.a | stressful relationship with parents, problems faced in school, stressful peer relationships, stress for future planning |
| 4 | Nair et al (2013) | Allapuzha, Kerela: cross-sectional | quantitative | semi-urban, rural, urban | 11 to 19; 537 | both male and female | Community-based | n.a | n.a | anxiety disorder, depressive disorder |
| 5 | Modi et.al (2015) | eastern india: cross-sectional | quantitative | n.a | 10 to 15; 62 | both male and female | Hospital-based | deliberate self-harm | organophosphorus oleander,  insecticide,  phenyl,  kerosene | Family stress, study stress, lower socio-economic status |
| 6 | Bindhani ( 2017) | Odisha; cross-sectional | mixed method | n.a | 10 to 15; 356 | both male and female | School-based | influenced suicide | n.a | admin tries to understand the mind of the player and then accordingly provokes suicide. |
| 7 | Aron et.al (2004) | Vellore,Tamilnadu; cross-sectional | quantitative | rural | 10 to 19; 306 | both male and female | Community-based | completed suicide | hanging, poisoning, self-immolation, drowning. | family conflicts, domestic violence, academic failures, unfulfilled romantic ideas, mental illness,easy availability of pesticides and poison . |
| 8 | Mathew & Nanoo (2013) | Kerela, case-control study | quantitative | urban | 13 to 19; 100 | both male and female | Hospital-based | attempted suicide | pesticide consumption, drug overdose,  odollum /nerium consuption, slashed wrist,  hanging ,  drowning,  burns  and consumption of unknown substance. | **Family history:** of mental illness, suicide attempt **Repeated suicide attempts** **Present attempt** **psychiatric morbidity:** adjustment disorder, depressive disorder, boderline personality disorder, conduct disorder with alcohol abuse, alcohol dependence syndrome and psychoses. **physical illness** **recent stresstressful event** |
| 9 | Sudhir et.al (2000) | Pudduchery; cross-sectional | quantitative | n.a | n.a; 74 | both male and female | Hospital-based | attempted suicide | organophosphorous, yellow oleander, prescription drugs overdose,  poisoning hanging, burning | **Family history** of: psychiatric disorder, substance abuse, attempted suicide, completed suicide. **Own history:** serious medical disease, alcohol abuse, psychiatric disorder, attempted suicide previously **depression** |
| 10 | Kumar et.al (2017) | Varanasi, UP cross-sectional | quantitative | both urban and rural | below 20; 340 | both male and female | Hospital-based | deliberate self-harm | n.a | Financial crisis, family quarrels, failure in examination, exposure to shameful events failure in love friend circle loss of near and dear scolding by parent/teacher personal illness |
| 11 | Jeypal et.al (2020) | South india; cross-sectional | quantitative | urban | 15 to 18; 8 | both male and female | Hospital-based | attempted suicide | yellow phosphorous | n.a |
| 12 | Verma et.al (2021) | Kota, Rajashthan; cross-sectional | quantitative | n.a | 10 to 19; 94 | both male and female | Hospital-based | completed suicide | hanging,  poisoning | sexual assault, love failure, unwanted marriage, unwanted pregnancy, less emotional support by family |
| 13 | Bhosle et.al (2015) | Nanded, maharashtra; longitudinal study | quantitative | urban | 10 to 19; 102 | both male and female | Hospital-based | completed suicide | hanging | domestic strife, examination-related stress, physical illness, psychological illness, failure in love, alcohol abuse |
| 14 | Sinha et. Al (2021) | UP and Bihar; cross-sectional | quantitative | both rural and urban | 10 to 19; 15388 | both male and female | Community-based | deliberate self harm | n.a | internet acess, parental abuse, involvement in fights, substance abuse and depressive symptomps |
| 15 | Beattie et al. (2019) | north Karnataka; cross-sectional | quantitative | rural | 13 to 14; 1191 | female | Community-based | NFSB (suicidal ideation) | n.a | sexual abuse, lack of parental emotional support |
| 16 | Kumar et. Al (2021) | UP and Bihar; cross-sectional | quantitative | both urban and rural | 13 to 19; 20700 | both male and female | Community-based | NFSB (suicidal ideation) | n.a | never attended or drop out from school, frequent media exposure, mother experienced physical abuse, had faced physical or sexual abuse, poor communication with parents, poor class performance used social media frequently, could not express opinions in family, had to confront wrong acts, who had any adult as role model, discussed about their relationship with other boys/girls, depression, |
| 17 | Sharma (2020) | Goalpara, Assam; cross-sectional | quantitative | urban and rural both | 16 to 19; 250 | both male and female | college-based | NFSB ( suicide ideation and plan) and suicide attempt | n.a | Psychological problem, family problem, physical problem, academic problem, negative peer relation, economic aspect, sexual abuse, problem related to love affairs, family history of substance abuse, family history of mental illness, socio-cutural factors, high risk behaviours |
| 18 | Sharma et.al (2019) | Goalpara, Assam; quasi-experimental study | quantitative | urban | 16 to 19; 80 | n.a | college-based | NFSB | n.a | psychological problem, family problem, physical problems, academic problems, negative peer relations, economic aspect, sexual abuse, problem related to love affair, family history of substance abuse, family history of mental illness, sociocultural factors, high-risk behaviour |
| 19 | Chandra et.al (2019) | Uttarakhand and Uttarpradesh; cross-sectional | quantitative | n.a | 15 to 18; 240 | both male and female | college-based | NFSB (suicidal ideation) | n.a | being a single child in the family, studying in non-co-education school, deviant parenting, |
| 20 | Bhan et.al (2019) | Bihar and U.P cross-sectional | quantitative | both rural and urban | 10 to 19; 20594 | both male and female | Community-based | NFSB (suicidal ideation) | n.a | witnessed parental marital violence, Child abuse, depression, personal experience of marital violence |
| 21 | Narain  (2021) | Patna; cross-sectional | qualitative | urban | 16 to 18; 106 | both male and female | School-based | NFSB (suicidal ideation) | n.a | lonliness, social alienation from family |
| 22 | S. Khurana et.al (2004) | Delhi; cross-sectional | quantitative | n.a | 10 to 16; 150 | both male and female | Child observation home (institution based) | suicidal behavior (thought, plans and attempt) | n.a | Abuse in family, |
| 23 | Thakur et. al (2015) | shimla, himachal pradesh; cross-sectional | quantitative | urban | 14 to 19; 218 | both male and female | School-based | NFSB (suicidal ideation) | n.a | low education status of mother, does not discuss problems with family, physical abuse |
| 24 | Sapna (2013) | Haryana, cross-sectional | quantitative | n.a | 14 to 17; 280 | both male and female | School-based | NFSB (suicidal ideation) | n.a | depression, academic self-afficacy |
| 25 | Manohar et.al (2016) | warangal; cross-sectional | quantitative | n.a | 13 to 18; 117 | both male and female | Hospital-based | completed suicide | n.a | Physical Illness , Failure in studies , Punishment , Failure in love ,  Domestic problems , Financial problems, Demand for Dowry  Premarital Pregnancy |
| 26 | Agarwal et.al (2019) | Kashmir; cross-sectional | quantitative | n.a | 16 to 18; 100 | only males | School-based | NFSB (suicidal ideation) | n.a | Physical aggression, Verbal aggression, Anger, Hostility |
| 27 | Nalawade et.al (2020) | Mumbai, Maharashtra cross-sectional | quantitative | n.a | 14 to 16; 120 | both male and female | School-based | NFSB (suicidal ideation) | n.a | Bullying |
| 28 | Parikh et.al (2019) | Goa and Delhi; cross-sectional | qualitative (FGD) | urban | 11 to 17; 191 | both male and female | both community and school-based | NFSB (suicidal ideation) | n.a | Sexual assault and rape, severe and sustained academic pressure |
| 29 | Manohar et.al (2016) | Warangal; cross-sectional | quantitative | Rural-urban and semi-urban | 13 to 18; 117 | both male and female | Hospital-based | Completed suicide | poisoning,  burns,  hanging,  drowning | Adoption of western lifestyle, addiction to the internet,  social networking and poor communal relationships. |
| 30 | Ahad et.al (2018) | Jammu & Kashmir; cross-sectional | quantitative | n.a | 15 to 18; 1069 | both male and female | School-based | suicidal ideation and attempt | n.a | n.a |
| 31 | Bhola et al. (2014) | Bangalore; cross-sectional | quantitative | urban, rural and semi-urban area | 16 to 18; 1087 | both male and female | college-based | suicidal ideation and attempt | n.a | gender (ideation), emotional difficulties (ideation), hyperactivity/ inattention, gender (attempt) |
| 32 | Bajaj et.al  (2020) | Punjab; cross-sectional | quantitative | urban | 12 to 17; 200 | both male and female | Community-based | NFSB (suicidal ideation) | n.a | Anxiety |
| 33 | Kaur et.al  (2020) | Ludhiana; cross-sectional | quantitative | both rural and urban | 16 to 18; 240 | both male and female | School-based | NFSB (suicidal ideation) | n.a | emotional stability, overall adjustment, autonomy, security-insecurity, self-concept, intelligence, |
| 34 | Bano et.al  (2019) | Gujrat; cross-sectional | quantitative | n.a | 12 to 19; 370 | both male and female | college-based | NFSB | n.a | n.a |
| 35 | Sharma et.al (2008) | South Delhi; cross-sectional | quantitative | n.a | 14 to 19; 550 | both male and female | college-based | suicidal behaviour | n.a | age group, gender living status of parents , working status of mother, working status of subjects role models seen smoking or drinking |
